# Managing cancer patients during COVID-19 pandemic: a North African oncological center experience

**DOI:** 10.11604/pamj.supp.2020.35.144.24582

**Published:** 2020-08-13

**Authors:** Youssouf Mohamed Ammor, Zinah Idrissi Kaitouni, Mouna Darfaoui, Issam Lalya, Abdelhamid Elomrani, Mouna Khouchani

**Affiliations:** 1 University Teaching Hospital Mohamed VI, Hematology and Oncology Center, Radiation Oncology Department, Marrakesh, Morocco

**Keywords:** COVID-19, cancer, prevention

## Abstract

**Introduction:**

since the first spread of the novel coronavirus (COVID-19) in Morocco in March 2020, the Moroccan Health System underwent an important pressure and remarkable efforts were spent to provide efficient reactions to this emergency. Public hospitals have set adapted strategies dedicated to overcoming the overload of COVID-19 patients, and our Oncology and Hematology Center (OHC) has implemented a flexible adapted strategy aiming to reduce the burden of COVID-19. We report our single-center experience on the detailed infection control measures undertaken to minimize virus transmission.

**Methods:**

we reviewed all patients treated at the OHC from March 2^nd^ to April 20^th^, 2020 as measures were taken since the detection of the first COVID-19 case to ensure the protection of patients and healthcare providers, especially a screening zone for any patient entering the center. The patient's data were retrospectively collected and anonymized.

**Results:**

we notified a significant decrease in patients' admissions during the lockdown period at the different units of our center. The screening area received a total of 5267 patients during our study period, with an average of 105 patients per day. Interestingly, no healthcare professional was infected and only 8 patients showed symptoms of fever and cough, and all of them had a negative test for COVID-19 (RT-PCR). Thus, the OHC is considered as a COVID-19 free center with zero cases among patients and healthcare providers.

**Conclusion:**

by having a 0% rate of infection, the efficiency of our measures is proven, but efforts are still needed as we have to measure the impact of this pandemic in our cancer management.

## Introduction

Since December 2019, the world has known the outbreak of a novel coronavirus named SARS-CoV-2 that first appeared in Wuhan (Hubei province, China). Since then, the spread of coronavirus disease 2019 (COVID-19) has progressively involved countries outside China leading the World Health Organization (WHO) to recognize it as a pandemic. It is therefore considered an emerging health threat (WHO, 2020) [1]. The COVID-19 pandemic was confirmed to have spread to Morocco on March 2^nd^, 2020 when the first case was reported [2]. Because of the virus´s rapid human-to-human transmission and potential severity, the Government of Morocco has announced by the 20^th^ of March a “Health State of Emergency” as part of its policy to limit the spread of the virus. Thus, a lockdown has been applied with restriction of movements within and between the cities along with sealing the national borders. During this crisis, the Moroccan Health System underwent an important pressure and remarkable efforts were spent to provide efficient reactions to this emergency. Public hospitals have set adapted strategies dedicated to overcoming the overload of COVID-19 patients. Currently, the Emergency Medical System is being implemented with material supplies such as respirators, personal protection equipment (PPE), and treatment. But also with healthcare providers. For this purpose, medical doctors from different departments and specialties were recruited to provide their assistance in managing patients suffering from COVID-19.

In this very unusual situation, cancer patients are considered as a highly vulnerable group and more susceptible to infections as compared to general population due to systemic immunosuppression secondary to both the malignancy and anticancer treatments. In the literature, Gwan *et al*. were the first to focus on oncological cases affected by COVID-19, through their study they concluded that the risk of SARS-CoV-2 infection was higher in cancer patients deteriorating more rapidly and presenting higher risks of severe events including the necessity for admission to the ICU and death. Interestingly, older adults and patients with pre-existing comorbidities (commonly diabetes and cardiovascular disease) are facing the most severe and critical consequences of the SARS-CoV-2 infection. Hence, the current emergency is of particular concern to medical oncologists, radiotherapists, and their patients [3]. The Oncology and Hematology Center (OHC) as part of the Mohamed VIth University Teaching Hospital of Marrakesh, Morocco has implemented a flexible adapted strategy aiming to reduce the burden of COVID-19. Our infection control protocol consisted of three measures: To enhance safety measures for both patients and workers in the center, to prioritize and adapt treatment protocols, and to provide appropriate care without delays for urgent patients. In the present paper, we report our single-center experience on the detailed infection control measures that were undertaken to minimize virus transmission between cancer patients and between the patients and the healthcare workers. Measures entailing screening of suspect cases, reorganization of the treatment facility, and protection of patients and healthcare workers were described. With our infection control protocol, we recorded zero COVID-19 cases among the patients and healthcare workers between March 2^nd^ and April 20^th^, 2020.

## Methods

**Patients' cohort:** we reviewed all patients who were treated at the Oncology and Hematology Center (OHC) of Mohammed VIth University Teaching Hospital of Marrakesh, Morocco, from March 2^nd^ to April 20^th^, 2020. The starting date of this study preceded the country lockdown by 18 days, as measures were taken since the detection of the first COVID-19 case to ensure the protection of patients and healthcare providers. Patient´s data such as medical history, contact with a COVID-19 positive patient, suspected symptoms and clinical examination, biological investigations, and possible chest imaging, were retrospectively collected and anonymized.

**Infection control measures:** strong infection control measures were implemented all over the OHC, including a new sorting area dedicated to patient screening, a special clinical unit to manage suspected COVID-19 cases, and adapted schedules for healthcare workers and treatment administration.

**Figure 1:**
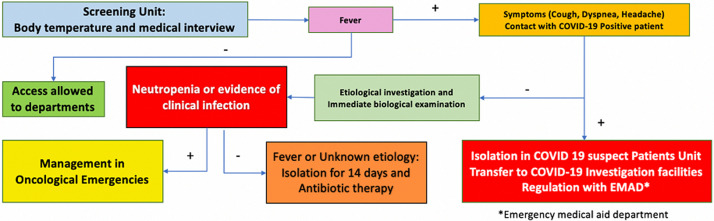
description of the reorganization of our hematological and oncological center with the establishment of sorting unit and individual pathways for patients along with a private entrance for medical staff

**Screening/sorting zone:** the sorting zone was an interface area situated at the entrance of the OHC. A strict and safe triaging procedure was performed to assess any COVID-19 symptoms as shown in Figure 1. Thus, all patients were checked for their body temperature before accessing the center followed by a quick check-up for COVID-19 symptoms such as cough, diarrhea and asthenia, and history of contact with SARS-Cov 2 infected patients. In the case of isolated fever, dedicated medical staff performed clinical and biological investigations in order to diagnose an underlying etiology. Suspected COVID-19 patients were isolated in a dedicated area, and then referred to COVID-19 facilities for further examinations. Non-suspected patients were given surgical masks and put under primary hygiene measures such as hands and accessories disinfection and shoes and wheelchairs cleaning using a carpet soaked with Sodium Hypochlorite.

**Figure 2:**
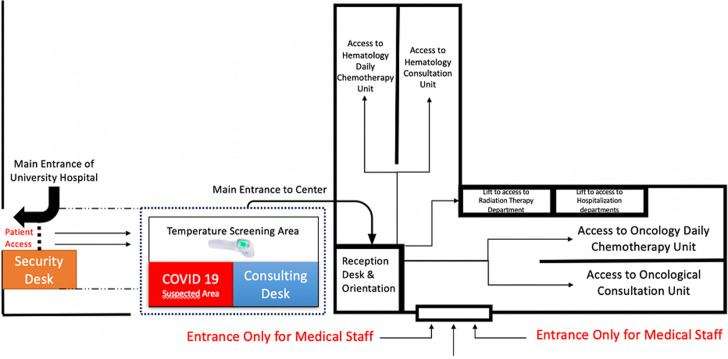
algorithm of cancer patients' management undergoing the sorting unit

**Organization measures:** during the COVID-19 pandemic, the units of OHC were managed to ensure both, the protection and good care delivery for all cancer patients as shown in Figure 2. For this purpose, several measures were established: 1) Entry/exit points were reduced, marking separately “staff” and “patients” entrances to facilitate communication and policy adherence. 2) The reception was shifted to the main entrance, and patients in wait were separated by a security distance of 2 meters using soil-markers. 3) Center attendance was limited to the patient and one visitor accompanying children and patients with special needs. 4) In waiting rooms, patients were educated to respect strict social distancing and avoid communicating with each other. Different educational posters and live TV broadcasts were displayed to highlight the importance of hygiene measures and distancing. The waiting room chairs were disinfected continuously. 5) OHC outpatient´s care Units were managed to receive a limited number of patients within specific daily time slots. Telemedicine was adopted to reduce in-person hospital visits during an infectious pandemic. 6) Hospitalization unit was managed to assure treatment for patients undergoing chemotherapy in individual rooms, equipped with disposable materials to prevent any risks of transmission. Moreover, rooms were disinfected on a daily basis. The prophylactic use of granulocyte colony-stimulating factor (G-CSF) was expanded from high risk to intermediate risk regimens in order to minimize risk of febrile neutropenia. 7) Ambulatory care hospital was supplied with 20 medical armchairs, distanced by 1.5 meters to ensure optimal distancing between patients, and all armchairs were disinfected between patients. The switch from intravenous chemotherapy to acceptable oral alternative was considered on a case-by-case basis as well as the use of 3 weeks protocols instead of weekly chemotherapy. 8) In Radiation therapy unit, the immobilization devices, CT scanner, and treatment tables were disinfected following each use. Patients´ overcrowding was limited by scheduling treatment sessions of 20-40 min for each patient and hypo-fractionated protocols were adopted for validated indications. 9) Finally, internal and multidisciplinary meetings were maintained using video-conference platforms. Protection of healthcare workers given the high risk of COVID-19 pneumonia for frontline medical staff, measures were implemented to mitigate the risk of cross-infection from patients to healthcare workers. Training sessions on the management of Covid-19 patients and self-protection were organized, the availability of Personal Protective Equipment (PPE) was assured following the WHO recommendations and healthcare providers were dispatched in two teams ensuring one shift every two days.

**Statistical analysis:** for descriptive analysis, continuous variables were presented as the mean with standard deviation or as median with interquartile range (IQR), as appropriate. Categorical variables are presented as percentages (%). The Shapiro-Wilk test was used to test the normality of data distribution.

## Results

### Cohort characteristics

All the demographic features included in the present study describe the period from March 2^nd^ to April 20^th^, 2020, and are summarized in Table 1, Table 2, and Table 3.

**Table 1:** demography and characteristics of patients admitted to the oncological department during COVID-19 outbreak

Consulting Unit	Data	Ambulatory chemotherapy Unit	Nb of Patients
New Cases	212	New Admissions	76
Median of Age	54 y.o.	Old Admissions	1160
Sex (Male/Female) %	42%/58%	Nb Chemotherapy sessions	1000
Old cases	956	**Hospitalization Unit**	**Nb of Patients**
**Tumour Diagnosis**	**%**	Sex (Male/Female)	115/103
Breast	26%	Mean Age	52 y.o.
Head and Neck	16%	**Tumour Diagnosis**	**%**
Gynaecological	13%	Head and Neck	68%
Thoracic	12%	Gynaecological	39%
Upper GI	10%	Thoracic	34%
Lower GI	10%	Upper GI	34%
Genitourinary	6%	Lower GI	22%
Sarcoma	3%	Genito Urinary	14%
Others	4%	Sarcoma	12%
Oncological Emergencies	192 patients	Breast	5%
		Nb Chemotherapy sessions	192

**Table 2:** demography and characteristics of patients admitted to the hematological department during COVID-19 outbreak

Consulting Unit	Data	Hematological Emergencies	121
New Cases	121	**Ambulatory chemotherapy Unit**	**Nb of Patients**
Median of Age	49	New Admissions	616
Sex (Male/Female) %	47%/53%	Old Admissions	60
Old cases	573	Nb Chemotherapy sessions	437
**Tumour Diagnosis**	**%**	**Hospitalization Unit**	**Nb of Patients**
Anaemic Syndrome	41%	Sex (Male/Female)	49/42
Leukaemia	19%	Mean Age	45
Lymphoma	7%	Nb Chemotherapy sessions	339
Others	33%		

**Table 3:** demography and characteristics of patients admitted to the radiation therapy department during COVID-19 outbreak

New cases Consultations	87	Surveillance consultation	1307
**Dosimetric CT scan Admission**	**167**	**Tumour Diagnosis**	**%**
**RT Treatment Admissions**	**142**	Breast Cancer	35%
**Mean patients treated per day**	**69**	Head and Neck	22%
Median of Age	51	Metastasis	14%
Sex (Male/Female)	30%/70%	Gynaecological	13%
**RT Strategies**	**%**	Genito Urinary	4%
Adjuvant	47%	Upper GI	4%
Definitive	39%	Lower GI	3%

**Figure 3:**
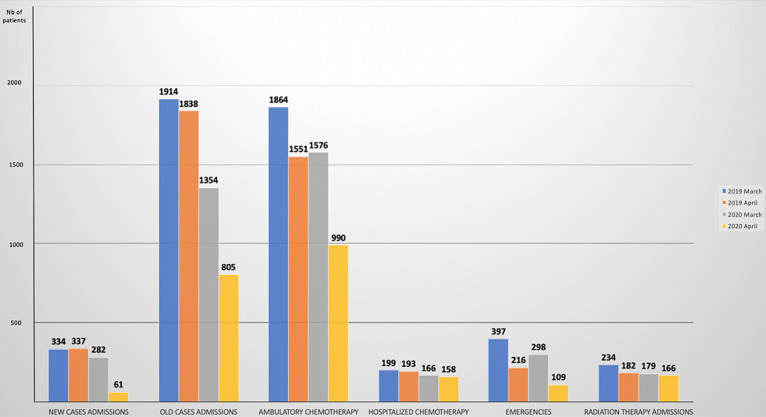
overall data of patients received in our center during the pandemic and compared to the same period of 2019

**Outpatient visits unit:** a total number of 343 new cases were admitted in the OHC, with 214 (62.3%) confirmed solid tumors and 103 (37.7%) hematological diseases. Telemedicine allowed us to reach 1440 patients mainly cancer survivors for surveillance visits. The median age was 54 years old (IQR: 15-88) for oncology patients and 49 years old (IQR; 1-83) for hematology patients. Gender was evenly distributed. Interestingly, we reported a decrease of 40% of newly admitted cases compared to the same period of the year 2019 (Figure 3).

**Emergency unit:** a total of 314 emergency cases (194 oncology patients and 120 hematology patients) were managed since the beginning of the pandemic. The median age was 68 and 45 years old respectively (IQR: O: 31-77, H: 11-66). In the oncology emergencies, 20% of patients required a blood transfusion, 20% received intravenous antibiotic treatment while 4% needed ascites or pleural evacuation puncture. In the Hematological Unit, the patients needed mainly blood transfusion (70%).

**Ambulatory care unit:** we had a total of 1912 admissions (1236 in oncology and 676 in hematology) which represents a decrease of 24% compared to the March-April 2019 period (Figure 3).

**Hospitalization units:** we registered a total of 228 admissions for Chemotherapy with a median age of 52 years old (IQR: 28-77). Head and Neck cancers (68 cases), gynecological cancers (39 cases), lung cancers (30 cases), and Upper gastrointestinal cancers (23 cases) were the most common localizations admitted to this unit. For the hematological department, we registered a total of 91 admissions and the median age was 45 years old (IQR: 17-66).

**Radiation therapy unit:** a total of 167 patients were admitted for CT-scanner simulation and 142 for treatment. The median age was 51 years old (IQR: 4-84). Breast cancer (35%), Head and Neck Cancer mainly nasopharyngeal and laryngeal cancer (22%), and Gynecological Tumors including Cervix cancer (14%) comprised the majority of the cases. Among these patients, 47% received Adjuvant RT, 39% received definitive RT while 14% received palliative treatments. Before and during the lockdown, the mean number of admitted patients per day was relatively constant (96 ± 3 patients).

### Outcomes

The screening area received a total of 5267 patients from March 2^nd^ to April 20^th^, 2020, with an average of 105 patients per day. Interestingly, only 8 patients showed symptoms of fever and cough, after deep investigations we report that: 1) One patient had a contact history with a confirmed COVID-19 case, the Rt-PCR result was negative and the patient displayed a normal chest CT scan. 2) One patient had tuberculosis with a negative Rt-PCR. 3) Two patients presented suspected CT scan profiles (ground-glass opacity) but the Rt-PCR results were negative. 4) Four patients underwent Rt-PCR for unexplained fever and respiratory symptoms but all came back negative. However, since those 8 patients were admitted mainly for chemotherapy, their treatment was postponed and they remained on quarantine for 14 days. Concerning healthcare providers, one staff member (A Radiation therapist) presented suspect symptoms including fever and cough with no previous contact history with a COVID-19 patient. He underwent an Rt-PCR and chest CT scan that came out negative. Thus, the OHC is considered as a COVID-19 free center with zero cases among patients and healthcare providers.

## Discussion

The present report describes the contingency plan established by the Oncology and Hematology Center (OHC) of Mohammed VI^th^ University Teaching Hospital of Marrakesh, Morocco. We shared our single-institution experience in the safety and management of cancer patients in the current severe acute respiratory syndrome coronavirus 2 (SARS-CoV-2) outbreak. Overall, the national COVID-19 situation is particularly under control in Morocco, this was challenging to achieve and to maintain for both national healthcare system and OHC which attracts patients from all the southern regions of the country. It is well established that due to their immunosuppressive state, cancer patients present higher vulnerability for infections [4-6]. This information was corroborated by the Liang *et al*. cohort describing that cancer patients had a higher risk of developing severe events (intensive care unit admission, invasive ventilation, or death) compared with general population (39% Vs 8%, p=0.0003). Moreover, according to the same study, patients who received antitumor treatment within 14 days before COVID-19 diagnosis, including, radiotherapy, systemic therapies, and surgery had a higher risk of developing severe events with borderline statistical significance [7]. Several other factors could account for an elevated risk for acquiring COVID-19 and serious complications in cancer patients, including frequent hospital visits and admissions, advanced age, and poor functional status [3].

Assuming these implications, several international guidelines were published concerning the management of cancer patients´ treatment during COVID-19 pandemic [8] along with oncological expert centers all around the world implementing drastic safety measures to ensure the protection of their patients and staff while guaranteeing the best care possible to their patients [9,10]. International experts´ recommendations shed lights on the importance of home management of cancer patients (e.g. telemedicine), as well as the replacement of intravenous drugs with adapted oral alternatives on a case by case basis (e.g., 5 Fluoro-Uracil and Capecitabine) [8]. Furthermore, the adjustment of dosing schedules of chemotherapy or radiotherapy treatments was recommended to reduce the frequency of hospital admissions (e.g., Paclitaxel every 3 weeks, rather than weekly administration and hypo-fractionated radiotherapy) [11]. OHC´s management strategy was in line with these international recommendations [11,12]. Our infection control protocol was covering a stepwise clinical pathway to manage suspect cases (symptomatic assessment, CT-scan, and RT-PCR assays). Many other measures were implemented, mainly on-site screening, re-organization of the OHC facilities, and protection of patients and healthcare workers.

Obviously, delayed or interrupted oncology treatments (e.g. surgery, chemotherapy, radiation therapy) may lead to disease progression and compromise survival outcomes. It was a major concern for us during this difficult period. We reported a diminishing number of outpatient visits and performed chemotherapy sessions, mainly because of the health emergency state and the inter-cities transport being suspended. It is mandatory, especially in the context of a limited resource system, to balance the potential benefit of containment measures, such as postponing scheduled procedures, with negative health and social costs. COVID-19 infection Diagnosis in cancer patients was particularly challenging, due to multiple factors. First of them would be the similarities of symptoms between the infection and the underlying disease, especially in lung cancer patients and patients with pulmonary metastasis which can hide an underlying COVID-19 infection causing a delay of diagnosis [13]. Adding to this, Cancer patients might have radiographic findings similar to those of a SAR-CoV-2 infection that can be misleading or paradoxically having atypical radiologic features of COVID-19 infection [14]. In our study, we conducted a systematic CT scan and RT-PCR to our suspected patients and due to the heterogeneity of radiological and biological findings, those patients were systematically put in quarantine and their cancer treatment was delayed. None of our patients or healthcare workers were infected which attests to the efficiency of our measures. We should point out that the actual global policy of diverting the attention exclusively to the COVID-19 pandemic and overshadowing other clinical conditions may have substantial negative implications for cancer patients and lead to an eventual spike of cancer deaths in Morocco within the next few months.

## Conclusion

Our main goal was to develop an adapted strategy to protect our patients and healthcare workers from contracting the novel coronavirus disease without impacting the quality of our healthcare services. By having 0% rate of infection, the efficiency of our measures is proven, but efforts are still needed as we have to measure the impact of this pandemic in our cancer management, and certainly more studies are yet to come to show this impact in our patients.

### What is known about this topic

- COVID-19 pandemic needed efficient screening protocols to prevent infected patients from harming other patients or healthcare professionals;
- According to a recently published Chinese cohort, patients with cancer had a higher risk of developing severe events (intensive care unit admission, invasive ventilation, or death) compared with patients without cancer (39% vs 8%, p=0.0003) (7);
- Cancer patients need continuous care, and interrupting their treatment in this outbreak could impact seriously their cancer prognosis, whereas their potential COVID-19 exposure could be very risky.

### What this study adds

- Even a developing country with limited health resources could have managed this outbreak by setting a good approach dealing with cancer patients during COVID-19 pandemic. Mainly with implementing an efficient screening protocol at the entrance of the center, and referring any suspected case to COVID-19 facilities;
- The main result was that our patients were well educated and informed about the risks of COVID-19 infection and how to prevent from it, and this leaded our center to be a free-COVID-19 facility during the first period of the country's lockdown.
